# Factors and Challenges in Increasing the Utilization Rate of a New Long-term Care Service (Kantaki) in a Superaging Society: Cross-sectional Study

**DOI:** 10.2196/45779

**Published:** 2023-05-09

**Authors:** Hiroshi Ono, Kuniko Haga, Eiko Nakanishi, Rika Watanabe, Masashi Manabe, Kenji Awamura, Takanori Kawano, Manabu Nii, Makiko Muya, Reiko Sakashita

**Affiliations:** 1 College of Nursing Art and Science University of Hyogo Akashi Japan; 2 Faculty of Nursing Tokyo University of Information Sciences Chiba Japan; 3 Faculty of Business Innovation Kaetsu University Kodaira Tokyo Japan; 4 Graduate School of Engineering University of Hyogo Himeji Japan

**Keywords:** aging, independent living, community health services, Kantaki, long-term care, terminal care, utilization rate, utilization, Japan, aging society, nursing, nursing services, home care, questionnaire, users

## Abstract

**Background:**

Japan is a superaging society unparalleled in the world. Elderly people who need medical care do not receive adequate support in the community. As a new service to address this issue, a small-scale multifunctional in-home care nursing service called Kantaki was created in 2012. Kantaki, in collaboration with a primary physician, operates 24 hours a day and provides various nursing services (home visits, home care, day care, and overnight stays) to older people living in the community. The Japanese Nursing Association is working hard to promote this system; however, its low utilization rate is an issue.

**Objective:**

This study aimed to determine factors influencing the utilization rate of Kantaki facilities.

**Methods:**

This was a cross-sectional study. A questionnaire on the operation of Kantaki was sent to all administrators of Kantaki facilities operating in Japan from October 1 to December 31, 2020. A multiple regression analysis was used to determine factors associated with a high utilization rate.

**Results:**

Responses from 154 of the 593 facilities were analyzed. The average utilization rate for all valid responding facilities was 79.4%. The average number of actual users and the break-even point were almost equal, resulting in little surplus profit from facility operations. A multiple regression analysis showed that factors that had a significant impact on the utilization rate included the break-even point, a surplus of users relative to the break-even point (ie, the margin of revenues), the number of months in office of the administrator, the type of corporation (ie, nonprofit), and Kantaki’s profit from operating home-visit nursing offices. The break-even point, a surplus of users relative to the break-even point, and the number of months in office of the administrator were robust. In addition, support for reducing the burden on family helpers, a service sought by the system, significantly and negatively affected the utilization rate. In the analysis that removed the most influential factors, the cooperation of the home-visit nursing office, Kantaki’s profit from operating the home-visit nursing office, and the number of full-time care workers were significantly related.

**Conclusions:**

To improve the utilization rate, managers need to stabilize their organization and increase profitability. However, a positive relationship was found between the break-even point and utilization rate, suggesting that simply increasing users did not contribute to cost reduction. Moreover, providing services that meet the needs of individual clients may result in lower utilization rates. These results, which are inconsistent with common sense, reflect the divergence between the assumptions underlying the system’s design and actual conditions. To solve these issues, institutional reforms, such as an increase in nursing care fee points, may be necessary.

## Introduction

### Background

Japan is the most aging society in the world, with 27% of the population aged >65 years in 2015; this figure is expected to increase to 40% by 2050 [1]. As the population ages, Japan’s mortality setting has changed dramatically. In 1952, 9.7% of deaths occurred in hospitals and 81.3% at home; however, in 2006, 79.7% occurred in hospitals and 12.2% at home [2].

The Japanese government launched a long-term care (LTC) insurance scheme in 2000, with the aim of enabling older people to live independently in the community. Under this system, individuals or their family apply for coverage with an insurer, usually the municipal government; individuals deemed to require long-term care are classified into 1 of 5 levels based on their level of dependence and their eligibility to receive services [3]. In this system, many home-care services provide support to older people in their home life. However, many of the staff providing these services are not qualified nurses, and older peoples’ medical care needs are not being met [4]. Visiting nurse services, which can provide medical care under LTC insurance, also face difficulties in providing continuous and sufficient medical support, as their activities focus on routine care with regular visits within a time limit [5]. As a result, older people who need medical care cannot receive adequate support in their communities and must be readmitted to the hospital. Rehospitalization attributed to the inability to continue care at home is due to a limited number of caregivers with an ability to care for older people with a deteriorating condition [6]. Many primary caregivers who live with older individuals requiring a high level of care are forced to leave their jobs and are involved in caregiving throughout nearly the entire day. In addition, most of those involved in such care are women [4]. This problem of maintaining life support for older people in need of medical care will become a global challenge in the future.

### Prior Work

In 2012, a service called Kantaki (the full name in Japanese is Kango Syokibo Takinou Kyotakugata Kaigo, which means “small-scale multifunctional in-home nursing care”), was established in Japan to provide community-based comprehensive care that allows the older population to live in the community [7]. The Ministry of Health, Labour and Welfare describes the service as small scale because it limits the number of users a facility can serve to 29 [8]. As a community-based service, Kantaki combines home-visit, day-care, and short-stay services to provide integrated services over a 24-hour period to older people who require nursing care while living at home [8]. By providing all these services from a single office in collaboration with a primary care physician, integrated and detailed care is possible. Kantaki is a groundbreaking service that leverages nursing expertise to improve residents’ quality of life.

Kantaki is expected to include end-of-life care support, medical support for patients with intractable diseases and dementia, support for reducing the burden on family caregivers, and utilization of community resources [8-10]. The Japanese Nursing Association [11] is promoting this service as a priority policy. Although there are 12,000 home-visit nursing offices nationwide, only 500 Kantaki facilities are available [12]; they are thus not sufficiently widespread. According to the results of interviews conducted by Mitsubishi UFJ Research and Consulting [9], the reasons for the lack of widespread use include the operational and staffing systems required to provide complex new services and challenges for users to understand and use the services offered. Consequently, many Kantaki facilities operate at loss, as they cannot secure the number of users [9]. Generally, facilities with financial challenges would either reduce their level of care to the poor and uninsured or face closure, bankruptcy, or merger [13]. In particular, smaller-sized facilities are less well managed than larger-sized facilities, making it difficult for them to provide services [4].

Fukui et al [14] conducted a study on the profitability of home-visit nursing offices in Japan using a questionnaire with 7 categories: operating structure, management by a nurse manager, employment, patient use, quality control, regional cooperation, and financial condition. The number of nursing staff, the number of users, being owned by a hospital, control of staff goals by nursing managers, and income compensation were reported as factors that increased profitability. Several studies of hospitals and nursing homes have reported that a higher utilization rate is related to better financial performance [15,16]. Lower occupancy has been found to be a significant predictor of financial problems [13]. A low utilization rate results in high operating expenses per client, which hinders efficient operations [13,17]. To stabilize operations, increasing the utilization rate is important. However, it is unclear to what extent specific facility, staff, and service characteristics impact utilization rates.

### Goal of This Study

The purpose of this study is to determine specific factors involved in facility, staff, and service characteristics that affect Kantaki’s utilization rates.

## Methods

### Study Design and Participants

This was a cross-sectional study conducted with a mailed questionnaire. Participants were administrators at Kantaki facilities (n=593) licensed to operate by local governments as of March 31, 2020.

### Survey Items

Based on the results of previous studies [8-10,14,18], the following variables were selected as independent factors that may affect the utilization rate of Kantaki: (1) facility characteristics, such as corporation type, cooperation of the home-visit nursing office, Kantaki’s profit from operating home-visit nursing offices, the actual number of users, maximum user capacity, number of months in business, number of months in office of the administrator, ratio of users with needs at each level of care (1-5), maximum distance to a user’s residence, break-even point of users, and the surplus of users relative to the break-even point (ie, the margin of revenues); (2) staff characteristics, such as the number of full-time nurses and care workers, turnover rate of nurses and care workers, and training participation rate of nurses and care workers; and (3) service characteristics, such as support for end-of-life care at home, support for patients with intractable diseases, functional training to reduce care needs, support for patients with dementia, support for reducing the burden on family helpers, support for medically dependent users, and interaction with local residents and participation in local activities (evaluated on a 5-point Likert scale).

The break-even point in facility characteristics is the point at which the revenue generated by facility operations equals the cost of the resources consumed to generate it [19]. Additionally, the surplus of users relative to the break-even point is regarded as the surplus width of revenue.

### Utilization Rate Calculation

Kantaki’s maximum capacity is set at a maximum of 29 persons according to the requirements of the personnel standards of the Ministry of Health, Labour and Welfare. As for the opening requirements, at least one staff member must be assigned to every 3 users for daytime services and at least two staff members for home-visiting services. The maximum capacity varies depending on the number of staff members employed at the facility [7].

The utilization rate was the dependent factor and was calculated based on the number of actual users and maximum user capacity using the following formula: (number of actual users / maximum user capacity) × 100 (%).

### Data Collection

At the end of March 2020, we collected public information on all Kantaki facilities registered with the Ministry of Health, Labour and Welfare. Questionnaires were mailed to each facility. The collection period was October 1 to December 31, 2020. A database was created from the response forms returned during this period.

### Statistical Analysis

Missing data from the survey responses were eliminated from the database, and other valid responses were included in the analysis. Statistical analysis was conducted with SPSS (version 26; IBM Corp). For descriptive statistics, we calculated the median (range) and mean (SD) for each item, and then examined the relationship between the utilization rate and each factor using the Spearman rank-order correlation coefficient.

Subsequently, a multiple regression analysis was conducted to determine the relationship between the utilization rate and each factor. Dummy variables were created for corporation type, cooperation of home-visit nursing offices, and Kantaki’s profit from operating the home-visit nursing offices. The variance inflation factor (VIF) was used to avoid multicollinearity. We judged VIFs greater than 10 as representing multicollinearity and excluded them from the items. VIFs greater than 4 also raised the suspicion of multicollinearity; however, we retained those with high *P* values in the results to avoid the possibility of increasing the arbitrariness of the model [20]. All the variables were first established in the model and analyzed using the backward selection method. Regarding the size of the adjusted *R*^2^ value and VIF, high *P* value variables were removed sequentially. The α level was set to .05 for statistical tests. In model 1, the goal was to maximize the size of the adjusted *R*^2^ value. In model 2, we excluded items with a VIF greater than 4. Finally, in model 3, the high impact variables in model 1 were excluded, retaining those with *P* values below .05.

### Ethical Considerations

This study was approved by the Research Ethics Committee of the Research Institute of Nursing Care for People and Community, College of Nursing Art and Science, University of Hyogo (2019F25). We asked the administrators of the Kantaki facilities to participate in our research project and obtained their consent.

## Results

### Characteristics of the Data

We received responses from 193 of 593 facilities (for a collection rate of 32.5%). Of these 193 facilities, 154 (79.8%) provided the data required for calculating the utilization rate (ie, the actual number of users and facility capacity) and were thus included in the analysis.

Descriptive statistics and correlations between each item and utilization rate are shown in Tables 1 and 2. Some items had missing data. The average utilization rate for all valid responding facilities was 79.4%. The average number of actual users was 21.1, whereas the average break-even point was 20.9, resulting in little surplus profit from facility operations.

The tenure of administrators was shorter than the number of months the facility was in operation, with a maximum of 84 months. The percentage of users at each level of care was approximately 20%, and there was no bias. The number of full-time care workers was approximately twice as large as that of full-time nurses. Among the service characteristics, support for reducing the burden on family helpers was addressed with the greatest emphasis. The correlations between each independent factor and utilization rate were all *P*<.6, except for the number of actual users.

**Table 1:** Descriptive statistics and correlation with utilization rate of each item at the facilities (n=154).

Variables	Median (range)	Mean (SD)	Correlation coefficient with utilization rate^a^	*P* value
Utilization rate, %	81.4 (20.8 to 100)	79.4 (18.9)	1.000	
Actual users, n	22 (5 to 29)	21.1 (5.5)	0.774	<.001
Maximum user capacity, n	29 (8 to 29)	26.7 (3.8)	–0.224	.005
Months in business, n	42 (7 to 96)	46.7 (24.0)	0.086	.16
Months in office of the administrator, n	36 (0 to 84)	35.7 (21.2)	0.109	.18
Ratio of users with level 1 needs, %	13.7 (0 to 60)	17.0 (14.1)	–0.005	.95
Ratio of users with level 2 needs, %	18.8 (0 to 47.1)	19.5 (10.5)	0.033	.69
Ratio of users with level 3 needs, %	18.6 (0 to 72.2)	19.8 (10.8)	0.049	.55
Ratio of users with level 4 needs, %	18.9 (0 to 56.3)	20.5 (11.5)	–0.020	.81
Ratio of users with level 5 needs, %	18.9 (0 to 78.9)	23.2 (16.3)	–0.001	.99
Maximum distance to a user’s residence^b^, km	8.0 (0 to 45)	8.9 (6.4)	–0.150	.37
Break-even point of users^c^, n	20.5 (4 to 29)	20.9 (4.7)	0.419	<.001
Surplus of users relative to the break-even point^d^, n	0 (–13 to 11)	–0.1 (4.1)	0.513	<.001
Number of full-time nurses, n	3 (0 to 10)	3.9 (2.1)	–0.014	.86
Number of full-time care workers, n	7 (1 to 14)	7.5 (3.1)	0.243	.002
Turnover rate of nurses^e^, %	0 (0 to 100)	12.6 (19.0)	0.035	.67
Turnover rate of care workers^f^, %	0 (0 to 133.3)	14.3 (22.5)	–0.105	.20
Training participation rate of nurses^g^, %	44.4 (0 to 237.5)	54.0 (43.1)	0.031	.73
Training participation rate of care workers^h^, %	33.3 (0 to 153.8)	41.8 (33.6)	–0.015	.86
Support for end-of-life care at home, score	5 (1 to 5)	4.3 (0.9)	0.138	.09
Support for patients with intractable diseases^f^, score	4 (1 to 5)	3.8 (1.0)	0.117	.15
Functional training to reduce the care need, score	4 (1 to 5)	3.9 (0.9)	0.012	.88
Support for dementia patients, score	4 (2 to 5)	4.3 (0.7)	0.082	.31
Support for reducing the burden of family helper, score	5 (3 to 5)	4.6 (0.6)	0.019	.81
Support for medically dependent users, score	5 (2 to 5)	4.4 (0.7)	–0.010	.91
Participation in local activities, score	4 (1 to 5)	3.5 (0.9)	0.145	.07

^a^Spearman rank test.

^b^143 facilities.

^c^138 facilities.

^d^137 facilities.

^e^152 facilities.

^f^153 facilities.

^g^123 facilities.

^h^133 facilities.

**Table 2:** Descriptive statistics and correlation with utilization rate of dummy variables at the facilities (n=154).

Dummy variables			Dummy variable=yes (facilities), n		Average utilization rate, %		Dummy variable=no (facilities), n		Average utilization rate, %		*P* value^a^		Correlation coefficient with utilization rate^b^
**Type of corporation**													
	For profit	53		79.9		101		78.4		.81		–0.020	
	Medical	43		80.1		111		77.5		.35		–0.076	
	Social welfare	35		78.2		119		83.2		.14		0.122	
	Nonprofit	5		79.3		149		82.8		.56		0.045	
	Other	18		79.5		136		78.2		.55		–0.049	
Cooperation of home-visit nursing office			36		80.0		118		77.5		.78		–0.023
Kantaki’s profit from operating home-visit nursing offices			31		79.0		123		80.8		.51		0.053

^a^Mann-Whitney *U* test.

^b^Spearman rank test.

### Impact of Each Factor on the Utilization Rate

The results of a multiple regression analysis with the utilization rate as the dependent variable are shown in Table 3.

**Table 3:** Factors related to the utilization rate.

Variable			β		Standardized β coefficient (95% CI)		*t^a^*		*P* value		VIF^b^
**Model 1 (n=109 facilities, adjusted *R*^2^=0.776)**											
	Constant	36.914		—^c^ (6.917 to 56.911)		3.875		<.001			
	Break-even point of users	2.807		0.687 (2.40 to 3.214)		13.715		<.001		1.212	
	Surplus of users relative to the break-even point	3.286		0.664 (2.807 to 3.765)		13.627		<.001		1.145	
	Number of months in office of the administrator	0.126		0.132 (0.037 to 0.214)		2.814		.006		1.059	
	Type of corporation (nonprofit)	17.930		0.151 (6.481 to 29.377)		3.108		.002		1.141	
	Cooperation of home-visit nursing office	–6.646		–0.150 (–16.013 to 2.721)		–1.408		.16		5.448	
	Kantaki’s profit from operating home-visit nursing offices	10.503		0.221 (0.443 to 20.564)		2.072		.04		5.481	
	Ratio of patients requiring level 3 care	0.107		0.062 (–0.0636 to 0.278)		1.246		.22		1.190	
	Turnover rate of care workers	–0.067		–0.069 (–0.166 to 0.031)		–1.360		.18		1.239	
	Training participation rate of nurses	0.008		0.020 (–0.034 to 0.052)		0.413		.68		1.164	
	Support for reducing the burden of family helpers	–3.988		–0.113 (–7.782 to –0.195)		–2.087		.04		1.406	
	Support for medically dependent users	–1.308		–0.049 (–4.362 to 1.747)		–0.850		.40		1.616	
**Model 2 (n=137 facilities, adjusted *R*^2^=0.661)**											
	Constant	16.824		—^c^ (6.467 to 27.182)		3.213		.002			
	Break-even point of users	2.591		0.634 (2.171 to 3.012)		12.19		<.001		1.083	
	Surplus of users relative to the break-even point	3.147		0.678 (2.671 to 3.623)		13.08		<.001		1.077	
	Number of months in office of the administrator	0.092		0.102 (0.001 to 0.183)		1.990		.049		1.047	
	Type of corporation (nonprofit)	8.738		0.087 (–1.412 to 18.888)		1.703		.09		1.040	
	Ratio of patients requiring level 3 care	0.221		0.129 (0.050 to 0.393)		2.554		.01		1.018	
**Model 3 (n=154 facilities, adjusted *R*^2^=0.091)**											
	Constant	68.425		—^c^ (60.451 to 76.398)		16.955		<.001			
	Cooperation of home-visit nursing office	–21.524		–0.484 (–37.809 to –5.238)		–2.611		.01		5.783	
	Kantaki’s profit from operating home-visit nursing offices	23.577		0.502 (6.429 to 40.274)		2.717		.007		5.755	
	Number of full-time care workers	1.500		0.244 (0.555 to 2.445)		3.137		.002		1.015	

^a^The *t* test was 2-tailed.

^b^VIF: variance inflation factor.

^c^Standardized β coefficient not calculated for constant.

### Analysis Including All Factors (Models 1 and 2)

Among the facility characteristics, nonprofit corporation type, number of months the administrator was in office, Kantaki’s profit from operating home-visit nursing offices, the ratio of patients requiring level 3 care, the break-even point, and the surplus of users relative to the break-even point affected the utilization rate. Among these characteristics, the break-even point of users, the surplus of users relative to the break-even point, and the number of months in office had a common influence in models 1 and 2 and were highly robust. In particular, the break-even point and surplus of users relative to the break-even point both had a standardized coefficient above 0.6 and had a high impact on the utilization rate. No significant factors were observed among the staff characteristics related to the utilization rate. Among the service characteristics, support for reducing the burden on family helpers had a negative impact on the utilization rate.

### Analysis Excluding High-Impact Factors (Model 3)

After excluding high-impact variables, the cooperation of home-visit nursing offices had a significantly negative impact and the profit from operating a home-visit nursing office and number of full-time care workers had a significantly positive impact on the utilization rate.

## Discussion

### Factors Influencing Kantaki’s Utilization Rate

While the surplus of users relative to the break-even point increased Kantaki’s utilization rate, the break-even point also had a very large impact on the utilization rate. Theoretically, the break-even point would be lower if services could be efficiently provided to a large number of users. However, this relationship was not observed in this study. A large break-even point indicates a high cost per user and low profit margin. Therefore, in order to sustain management, the facility administrators attempted to eliminate the deficit by increasing the number of users. Kantaki’s average labor cost accounted for approximately 75% of the total cost; the relative proportion of labor costs was high [9]. We presume that the large relative share of labor costs resulted in an increase in cost per user, leading to a positive correlation between the break-even point and the utilization rate.

Regarding staff characteristics, a greater number of full-time care workers contributed to a higher utilization rate. In general, turnover among care workers is high [21]. Reasons for this include low wages, increased work stress, and decreased job satisfaction [22]. In Japan, low wages for men and old age for women have been reported as the main reasons for leaving care worker jobs [23]. If the organization can increase its profit margins and improve the working environment, the utilization rates could improve. This is consistent with the finding that additional profit from operating a home-visit nursing office and the number of full-time care workers had an impact on utilization rates in model 3. Kantaki’s maximum capacity is based on the number of employed staff members. Improving the financial situation without lowering the cost to maintain staff is important for the expansion of Kantaki services.

Home-visit nursing offices cooperated in 23.4% (36/154) of the participating facilities but negatively impacted the utilization rate. This coincides with the results of a survey conducted by the Tokyo Metropolitan Government [24]. One of the advantages of running 2 facilities together is that nurses can work in both facilities at the same time [25]. However, nurses may not be exclusively involved in Kantaki, which requires integrated services and continuous support. It is important not only to have enough nurses to meet staffing standard requirements but also to make a sufficient contribution to Kantaki services.

### Challenges to Ensure the Stable Operation of Kantaki Facilities

In this study, the number of months in the office of the administrator affected the utilization rate. Nursing home administrators in the LTC sector are under great stress due to high work complexity and administrative responsibilities resulting from public policies and high-level specialization and competitiveness [26]. Administrator turnover is associated with poor quality of care and high turnover of care workers [21,27,28]. Therefore, reducing the turnover rate of administrators in the Kantaki facilities is essential to expand this service.

The surplus of users relative to the break-even point and Kantaki’s profit from operating the home-visit nursing offices contributed to the utilization rate. This highlights the importance of proper revenue generation in expanding services. However, although the average utilization rate was 80%, the average break-even point was not positive. We believe that poor profitability in the current system is one of the main issues that must be addressed.

The analysis of the service characteristics indicated that fulfilling the role required by the system did not improve the utilization rate. In addition, support for reducing the burden on family helpers had a negative impact on utilization rates, although it was the most important type of service for the administrators. This suggests that they might no longer be able to serve many individuals owing to the increased effort per user. Normally, facilities that provide better services to users should attract customers and thereby increase the utilization rate. With the aim of providing support to older people with medical needs to continue living at home, many nursing activities are conducted in Kantaki, including health management, determining the need for medical consultations, and emergency management [10]. Watanabe et al [18] reported that the average number of full-time equivalent staff was 13.4 for an average of 20.7 users. This number is much higher than the standard staffing requirement for a Kantaki facility. This indicates that labor costs are higher than the government assumed when the system was designed. Ensuring sufficient staff is important to maintain the quality of services [29]. However, under the current system, hiring staff beyond the standard staffing level does not result in an increase in facility revenue. In the future, it will be necessary to conduct a survey on the number of staff and the content and quality of services that can actually be provided, calculate compensation based on evidence, and reform the system to appropriately improve the utilization rate.

Government support in terms of funding and legislation is critical to the success of aging in place [30]. Supporting the lives of older people with medical needs requires a large number of staff and financial support to ensure that income is sufficient to meet necessary staff costs. Life support for older people who need medical care will become a global issue in the future. As the Kantaki system was established to support the people’s lives in the community, institutional improvements must be made to expand this small-scale, multifunctional in-home care service. The establishment and wide recognition of this service may be an effective strategy to provide end-of-life care support to older people in the community.

### Limitations

In this survey, complete data from only 154 of 593 (26%) facilities were included in the analysis. This inevitably resulted in bias. However, compared to the results of the study by Watanabe et al [18], which reported complete data on all facilities, no deviations in the ratio of corporation type or the required care level were observed. In addition, because this was a cross-sectional study, the data for each facility may reflect a temporary situation. A longitudinal study is required for a more detailed analysis.

### Conclusions

The break-even point of users and the surplus of users relative to the break-even point strongly impacted the utilization rate of the Kantaki facilities. The utilization rate was higher with a higher break-even point and an increase in the surplus of users relative to the break-even point. Long-term and stable efforts by administrators were also influences on a higher utilization rate. In contrast, providing services that could improve the quality of life of Kantaki users reduced the utilization rate.

To increase Kantaki’s utilization rate, facilities should increase profitability while maintaining employment levels. However, in reality, the break-even point can only be exceeded by increasing the occupancy rate to 80%. To expand this service, revising the system will be necessary to improve the profitability of facilities, such as by raising nursing care fee points.

## Abbreviations

LTC: long-term care
VIF: variance inflation factor
